# Interoception and sexual response in women with low sexual desire

**DOI:** 10.1371/journal.pone.0185979

**Published:** 2017-10-11

**Authors:** Julia Velten, Lori A. Brotto

**Affiliations:** 1 Mental Health Research and Treatment Center, Ruhr-Universität Bochum, Bochum, Germany; 2 Department of Obstetrics and Gynaecology, University of British Columbia, Vancouver, BC, Canada; Radboud Universiteit, NETHERLANDS

## Abstract

Sexual concordance is defined as the association between genital response and self-reported sexual arousal. Though one might predict a strong association between sexual concordance and awareness of other internal physiological sensations (termed *interoception)*, past research on sexually healthy women has not found these different domains to be related. The aim of the present study was to test the association between interoception and sexual concordance in a clinical sample of women with Sexual Interest/Arousal Disorder (SIAD). Fifty-two women with SIAD completed the Multidimensional Assessment of Interoceptive Awareness (MAIA), a validated self-report measure of interoception, and completed a heart-beat accuracy test, an objective measure of interoception. They also participated in a laboratory-based assessment of physiological sexual arousal and self-reported sexual arousal while viewing an erotic film. Mental and physiological arousal were correlated at *r* = 0.27 (range -0.80 to 0.95). There was no significant association between sexual concordance and women’s heartrate awareness. However, five aspects of interoceptive awareness (noticing, emotional awareness, self-regulation, body-listening, and trusting), were predictive of *lower*, and one aspect (not-distracting) was predictive of *higher* sexual concordance. We discuss the findings in relation to the role of emotions and arousal states in the interoception-sexual concordance relationship.

## Introduction

A sexual dysfunction arises when there is a disturbance in a person’s ability to respond sexually or to experience sexual pleasure, and it is associated with significant personal distress [1]. One of the most common sexual complaints in women is a lack of interest in sex, which is defined in the 5^th^ edition of the Diagnostic and Statistical Manual of Mental Disorders (DSM-5) as Sexual Interest/Arousal Disorder (SIAD). The diagnosis of this disorder requires at least three of the following: (a) a lack of interest in sex, (b) few/no sexual thoughts, (c) lack of receptivity to a partner’s sexual invitations and no initiation of sexual behavior, (d) a lack of pleasure during sexual activity, (e) lack of responsive desires to erotic triggers, and (f) reduced physical signs of sexual arousal [1].

Mindfulness has arisen as a promising approach to improving women’s low sexual desire [2–6]. By increasing women’s ability to attend to sexual stimuli, mindfulness may improve sexual function by directly increasing their sexual response [7]. Sexual response is generally thought of as an emotion with subjective or experiential, physiological, and behavioral components [8].

### Emotions and response coherence

Emotions have been defined as:

a complex set of interactions […] which can (a) give rise to affective experiences such as feelings of arousal, pleasure/displeasure; (b) generate cognitive processes […]; (c) activate widespread physiological adjustments to the arousing conditions; and (d) lead to behavior that is often, but not always, expressive, goal-directed, and adaptive [9].

According to influential theories, a defining feature of emotions is response coherence, such that different aspects of the emotion are correlated with one another [10–12]. The extent to which these response levels agree, differs between different emotions [13,14]. Research on anxiety and fear, for example, has revealed that physiological, experiential, and behavioral responses towards feared stimuli often occur at different time points and are at least partially independent [15–18].

Though emotion theory suggests that bodily sensations and other visceral responses are key to determining subjective emotional experience [19], there is substantial evidence in sexual psychophysiological research for a lack of response coherence (or concordance) between subjective and physiological responses [20]. Increased heart rate, increased blood pressure, and increased skin conductance levels have been described as physiological responses to visual erotic stimuli in some [21,22], but not in all studies [23]. The component of physiological arousal that is most specific to sexual arousal is the genital arousal response that includes increased blood flow to the genitals, resulting in a swelling of the clitoris, vagina, and vulva as well as vaginal lubrication in women [24,25].

In a meta-analysis of 132 studies that examined sexual arousal concordance (i.e., association between psychophysiological sexual arousal and self-reported sexual response), Chivers et al. [20] found concordance estimates in women to be consistently lower than concordance in men (*r* = .26 vs *r* = .62, respectively). To further understand this sex difference, they examined a large number of variables proposed to be related to concordance, such as: age, use of oral contraceptives, statistical measures, and type of sexual stimuli used. A statistically significant gender difference in sexual concordance was found regardless of most methodological factors. Understanding the nature of this sex difference in concordance has been of significant interest to sex researchers over the past decade.

In addition to this sex difference, Chivers et al. [25] found that women with sexual dysfunction had an average concordance score of *r* = .04, ranging from *r* = -.10 to *r* = .17, which was significantly lower than samples of sexually healthy women. The clinical significance of this low level of sexual concordance is unknown. Moreover, the association between sexual arousal concordance and other measures of response coherence has never been studied in a sample of women with sexual dysfunction, and is the aim of the present study.

### Interoceptive awareness and sexual concordance

Interoception refers to an individual's ability to recognize internal physiological states, such as heart rate or respiration rate. Some have speculated that the sex difference in sexual concordance may be due to a sex difference in overall interoceptive ability, such that the same processes that contribute towards awareness of physiological signs in the body may also contribute to awareness of signs of sexual arousal [26,27]. Men are generally more accurate than women at detecting their own heart rate [28], blood pressure [29], and stomach contractions [30]. This sex difference has been hypothesized to be due to a sex difference in emotional processing: in laboratory settings, men’s interoceptive abilities seems to be dependent on internal cues, whereas women’s interoceptive abilities are more reliant on external cues [20,31].

A few studies sought to explore the relationship between interoceptive awareness and sexual arousal concordance. In one study, healthy men and women viewed sexual and neutral film clips, during which their genital response, heart rate, and respiration rate were measured, using a vaginal or penile plethysmograph, electrocardiogram (ECG), and a thermistor [27]. After each clip, participants answered questions regarding their feelings of genital sexual arousal, their overall sexual arousal, their emotional experience, as well as their perceived heart rate and respiration rate. Although men showed higher concordance for genital response and heart rate than women, there was no association between sexual concordance and heart rate or respiration rate concordance. Since participants rated their heart rate and respiration rate awareness after the films, Suschinsky and Lalumière hypothesized that their ratings may have been influenced by memory or distraction from film clips [27]. Therefore, in a follow-up study, Suschinsky and Lalumière [32] utilized the Heartbeat Perception Task [33] to measure physiological awareness, which involved women counting their heartbeats before and after watching an erotic film clip, in 16 anxious and 15 non-anxious women. Again, sexual concordance was not significantly related to heartbeat perception accuracy or to respiration rate awareness in the combined group. Moreover, heart rate awareness and respiration rate awareness were also not significantly correlated. Suschinsky and Lalumière concluded that low levels of concordance may reflect the norm for women, but urged other researchers to explore this further in women with sexual dysfunction, given that anecdotal evidence suggests that a low awareness of physical sensations may be linked to low sexual desire in women [27].

To our knowledge, only one study has investigated the relationship between interoception and sexual concordance in women using a self-report questionnaire. In a sample of 26 women, the noticing subscale of the Multidimensional Assessment of Interoceptive Awareness scale (MAIA) [34] was related to greater sexual concordance. In other words, women who reported a greater awareness of uncomfortable, comfortable, and neutral body sensations showed a greater agreement of genital and subjective sexual arousal. Two limitations reduce the generalizability of their findings: The sample consisted of young, sexually healthy, college students. Therefore, findings may not apply to older women or women with sexual concerns. In addition, the MAIA was administered *after* a laboratory session that included a sexual arousal measurement including the instruction to focus attention on genital sensations. This procedure may have lead to higher scores on the MAIA and might have directly contributed to the association between laboratory-based MAIA scores and laboratory-based arousal concordance.

In the current study, subjective and genital arousal were assessed continuously during stimulus presentation [35]. Women’s genital sexual response was measured with a vaginal photoplethysmograph, a tampon-shaped device that measures vaginal blood flow. Vaginal pulse amplitude (VPA) is a valid and reliable measure for female genital arousal [8,36,37], although there is an ongoing scientific debate about its relationship to other aspects of women’s sexual response such as lubrication or clitoral blood volume [38–40]. Nevertheless, the usefulness of the VPA measure has been shown in a large body of research [20,41].

Taken together, the goal of this study was to examine the relationship between interoceptive awareness (measured both objectively and with a self-report measure) and sexual concordance in women with SIAD. We aimed to replicate the design from one previous study [32], but with two modifications: (a) we included a validated self-report measure of interoceptive awareness in addition to measuring heartbeat perception accuracy objectively; and (b) we applied the design to a sample of women with a diagnosed sexual dysfunction. Though we predicted a positive but small association between genital and subjective sexual arousal, we did not have a priori predictions about the association between sexual concordance and interoception, measured either objectively or subjectively, given conflicting findings in the literature to date. Given that our sample was relatively homogeneous in that they all met criteria for SIAD, we did not predict many individual differences in the associations between concordance and interoceptive awareness. A better understanding of the associations between awareness of interoceptive sensations and sexual response may shed light on the mechanisms by which psychosocial interventions, especially those aiming to increase awareness of the body, lead to improvements in women’s sexual functioning.

## Method

### Participants

Participants were part of a larger study evaluating outcomes of group mindfulness-based sex therapy on sexual desire, sexual response, and affect [5], though we only used the pre-treatment data in the present analyses. Women seeking treatment for sexual desire and/or arousal concerns and who lived in a large metropolitan city were eligible to participate. Inclusion criteria were: age between 19 and 65 years, fluent in English, willingness to participate in group sessions and to complete daily homework, and ability to participate in pre- and post-treatment assessments (including both self-report questionnaires and in-laboratory psychophysiological sexual arousal assessments) at three time points. Women with difficulties in achieving orgasm were also included as long as those were not experienced as more distressing than the desire and/or arousal concerns. We excluded any woman with dyspareunia (chronic genital pain not resolved with a personal lubricant). Fifty-two women, between the ages of 22 and 65 (*M* = 41.92 years, *SD* = 12.10) participated in the study. The average relationship length ranged from 0 to 37 years (*M* = 9.89 years, *SD* = 1.61), and the average duration of low desire ranged from 0.8 to 38.0 years (*M* = 8.72, *SD* = 9.23). A total of 78.8% of the women identified as heterosexual, 11.5% as bisexual, and 9.6% as lesbian.

### Audiovisual stimuli

Experimental stimuli consisted of a one-minute “relax” period, a five-minute neutral video, and a 10-minute erotic film. Erotic footage was standardized to include three-minutes of foreplay (story line, kissing, touching, undressing), three minutes of genital stimulation/oral sex, three minutes of vaginal penetration activities, and one minute of a wind-down/cuddle period. The film clip shown during the study was randomly selected by the researchers from a collection of similar erotic videos, but participants had a choice of either homosexual or heterosexual footage.

### Measures and instruments

#### Physiological sexual response

Vaginal pulse amplitude (VPA) was used as a measure of genital sexual response using a vaginal photoplethysmograph equipped with an orange-red spectrum light source (Behavioral Technology Inc., Salt Lake City, UT) during the experimental procedure. The signal was sampled at 200 Hz, band pass filtered (0.5–30 Hz), and recorded continuously during the stimulus presentation. Data were acquired and processed using a data acquisition unit Model MP150 and AcqKnowledge version 3.8.1 (BIOPAC Systems, Inc., Santa Barbara, CA). A trained research assistant visually inspected the data and performed smoothing of movement artifacts prior to data reduction and analysis.

#### Subjective sexual arousal

SSA was measured *continuously* during stimulus presentation with an arousometer that was constructed by a local engineer modeled after the one described by Rellini et al. [35]. The device consisted of a computer optic mouse mounted on a plastic track with 10 intervals, affixed to the armrest of the reclining chair. Women were instructed to use the arousometer continuously to indicate changes in mental sexual arousal from 7 (*highest level of arousal*) to 0 (*no sexual arousal*) and -2 (*sexually turned off*) during the entire duration of the erotic films. Similar devices have been used to assess subjective sexual arousal in previous laboratory studies [35,42].

#### Self-reported interoceptive awareness

The Multidimensional Assessment of Interoceptive Awareness (MAIA) survey was administered as a measure of interoceptive bodily awareness [34]. Questions are answered on a six-point Likert scale ranging from 0 (*never*) to 5 (*always*). It assesses eight dimensions of body awareness: noticing (awareness of uncomfortable, comfortable, and neutral body sensations), not-distracting (tendency not to ignore or distract oneself from sensations of pain or discomfort), not-worrying (tendency not to worry or experience emotional distress about sensations of pain or discomfort), attention regulation (ability to sustain and control attention to body sensations), emotional awareness (awareness of the connection between body sensations and emotional states), self-regulation (ability to regulate distress by paying attention to body sensations), body listening (active listening to the body for insight), and trusting (experiencing one’s body as safe and trustworthy). Cronbach’s alpha in the present sample were good (α > .80) for six of the MAIA subscales, acceptable (α = .72) for not-distracting, and questionable (α = .69) for the not-worrying subscale.

#### Objective measure of interoceptive awareness

A heartbeat perception task was used as an objective measure of interoceptive awareness [33]. The task consisted of three randomized trials of 25, 35, and 45 seconds, with 30-second rest periods between each trial. The beginning of each trial was indicated with the written words: “Count the number of heartbeats you feel in your body without taking your pulse or counting out loud” on the TV screen, after the researcher had left the room. After each trial, participants recorded their number of heartbeats as well as an estimate of how many they might have missed. We followed the procedures adopted by other researchers such that interoceptive heart rate awareness was calculated by taking the absolute difference between the reported number of heartbeats (sum of heartbeats that were felt and potentially missed) and the actual number of heartbeats (taken by the ECG) divided by the actual number of heartbeats in order to obtain an error score [32]. This value was then subtracted from one to obtain an interoceptive awareness score, and then averaged across the three trials [43].

### Procedure

An experienced research assistant with specialized training in the assessment and diagnosis of sexual dysfunctions conducted telephone screenings to assess the eligibility of potential participants. Following the telephone screen, she mailed a consent form to eligible women interested in participating. The assessment took place in a sexual psychophysiology laboratory, located at a university hospital. Following signed consent, participants were tested by a female researcher. The vaginal probe was attached to a plastic placement device which held the probe in a fixed orientation and depth. During the orientation phase, the researcher showed participants how to insert the vaginal probe, and which way to orient the placement device. This helped to ensure proper and consistent orientation and depth of the probe insertion across all participants [44]. Electrodes were then attached to the participant’s inner wrist and ankles before proceeding to another room, in which the experimenter was able to monitor heart rate to ensure the ECG was properly functioning.

Following this heartbeat perception task, the participant was then asked via intercom to remove the electrodes and proceed to insert the photoplethysmograph. Participants were also reminded to use the arousometer to capture their subjective sexual arousal throughout the erotic film presentation. The researcher instructed participants to monitor their subjective feelings of sexual arousal by using this device. She also explained that ‘subjective feelings of sexual arousal’ means how mentally sexually aroused the participant feels while watching the film.

The researcher then initiated the video sequence. Women watched a 5-min documentary followed by a 10-min erotic film that depicted a male-female couple engaging in foreplay, cunnilingus, fellatio, and penile–vaginal intercourse. They were then instructed to remove the probe and meet the researcher in a separate room. After a debriefing period, the researcher disinfected the probe in a solution of Cidex OPA (ortho-phthalaldehyde 0.55%), a high-level disinfectant (Advanced Sterilization Products, Irvine, CA, USA), promptly following each session. All procedures were approved by the Clinical Research Ethics Board at the University of British Columbia and the Vancouver Coastal Health Research Institute. All procedures were carried out in accordance with the provisions of the World Medical Association Declaration of Helsinki (2013).

### Data reduction and analysis

Off-line, plethysmography data were band-pass filtered (0.5–20 Hz). Then, in agreement with standardized procedures, movement artifacts, defined by sudden and drastic changes in pulse amplitude, were visually identified and deleted by being marked as missing for data analysis [36]. Both subjective sexual arousal and genital sexual arousal (in mV) were averaged across the erotic stimulus condition.

Data were analyzed using SPSS version 24 [45]. Because of the non-normality of the data, Kendal’s tau correlation coefficients between all predictor and outcome variables were calculated. A series of 18 multiple regression analyses were conducted to examine the relationship between interoceptive awareness and sexual arousal and concordance. Significant two-way interaction effects between one aspect of sexual response—subjective or genital sexual arousal—and a lower order scale of the MAIA were interpreted in a way that suggested that domain of interoceptive awareness significantly influenced sexual concordance (i.e., the relationship between subjective and genital arousal). When interactions were identified, post hoc simple slope analyses were conducted to determine if the slopes of the two predicted lines differed from zero [46]. In addition, within-subject-concordance of subjective and genital sexual response was calculated for each participant using a total of 20 30-sec bins during the erotic film segment of the film only.

## Results

### Descriptive analysis

Table 1 shows the descriptive values for interoceptive awareness and sexual response. During the total trial period of 105 seconds, the difference between participant’s actual heartbeats and their estimation was 38.7 heartbeats (*SD*: 20.8; Range: 4.29 to 100.0). Subjectively measured interoceptive awareness in our participants was significantly lower than those reported in the original MAIA validation sample [34]. Differences in interoceptive awareness between the two samples were medium (*d* = .51 for not-worrying) to large (*d* = 1.56 for self-regulation).

**Table 1:** Interoceptive awareness and sexual arousal measurements during erotic stimulus presentation.

		*Min*	*Max*	*M*	*SD*
Interoceptive awareness					
	Heartbeat perception task (accuracy score)	0.00	0.96	0.61	0.21
	Noticing (NC)	0.50	5.00	3.11	1.05
	Not-distracting (ND)	0.33	4.33	2.28	1.01
	Not-worrying (NW)	0.33	4.33	2.65	0.99
	Attention Regulation (AR)	0.00	4.29	2.33	1.03
	Emotional Awareness (EA)	0.80	5.00	3.23	1.06
	Self-Regulation (SR)	0.00	4.25	2.41	1.01
	Body-Listening (BL)	0.00	5.00	1.87	1.31
	Trusting (TR)	0.33	5.00	3.05	1.09
Sexual response					
	Subjective sexual arousal (Range: -2 to 7)	-0.18	6.90	1.58	1.40
	Genital sexual arousal (mV)	0.02245	0.12600	0.06150	0.02824
	Within-subject concordance	-0.80	0.95	0.27	0.49

Both genital sexual arousal (VPA), *t*(46) = -7.14, *p* < .001, and subjective sexual arousal, *t*(46) = -1.58, *p* ≤ .001, significantly increased between neutral and erotic film conditions suggesting that we were able to successfully elicit sexual arousal in our participants.

### Relationship between interoceptive awareness and sexual arousal and concordance

Heartbeat perception was not significantly correlated with any of the MAIA subscales or sexual arousal and concordance. Attention regulation was positively correlated with subjective sexual arousal, *r*_*τ*_ = .21 *p* = .031, and not-distracting showed a trend towards being positively correlated with subjective arousal, *r*_*τ*_ = .16, *p* = .096. In addition, emotional awareness was negatively correlated with genital sexual arousal, *r*_*τ*_ = .16, *p* = .067, though not significantly. None of the other subscales of the MAIA showed substantial bivariate correlations with sexual response or concordance. Table 2 shows the bivariate correlations between the facets of interoceptive awareness and sexual response.

**Table 2:** Nonparametric bivariate correlations (Kendal’s tau) between different sexual arousal measurements and interoceptive awareness.

				Multidimensional Assessment of Interoceptive Awareness (MAIA) scales								Sexual response		
			1	2	3	4	5	6	7	8	9	10	11	12
	Heartbeat perception task	1	1	.09	-.02	.11	.05	.12	.05	.05	.13	-.09	-.10	.13
MAIA	Noticing (NC)	2		1	.11	-.10	.43**	.57**	.41**	.45**	.34**	.00	-.13	.04
	Not-distracting (ND)	3			1	-.15	.15	.10	.08	.13	.06	.16^(*)^	-.13	.16
	Not-worrying (NW)	4				1	-.03	-.09	-.18	-.16	-.12	-.04	-.06	.01
	Attention Regulation (AR)	5					1	.48**	.54**	.57**	.53**	.21*	-.11	-.03
	Emotional Awareness (EA)	6						1	.48**	.54**	.39**	.03	-.18^(*)^	.04
	Self-Regulation (SR)	7							1	.62**	.45**	.13	-.12	.04
	Body-Listening (BL)	8								1	.49**	.02	-.11	.06
	Trusting (TR)	9									1	.11	-.02	.05
Sexual response	Subjective sexual arousal	10										1	.04	.18^(*)^
	Genital sexual arousal	11											1	.07
	Within-subject concordance	12												1

* *p* < .05

** *p* < .01

Relevant findings of the multiple regression analyses that have been conducted to assess the relationship between heartbeat perception (Independent variable, IV), self-reported aspects of interoceptive awareness (IV), and sexual response (dependent variables, DV) are presented in the following section.

An overview of the complete findings is presented in Table 3.

**Table 3:** Series of multiple linear regression analyses predicting genital (VPA) and subjective (SSA) sexual arousal.

		*VPA*		*SSA*	
		ß	*t*	ß	*t*
Heartbeat perception task					
	VPA/SSA	.14	0.97	.12	.89
	Heartbeat perception	-.11	-0.75	-.15	-1.08
	VPA/SSA * Heartbeat perception^a^	.23	1.61	.22	1.57
Noticing (NC)					
	VPA/SSA	.01	0.08	-.06	-0.44
	NC	-.14	-1.02	-.16	-1.26
	VPA/SSA * NC	-.31	-2.02*	-.55	-4.32***
Not distracting (ND)					
	VPA/SSA	.03	0.22	.30	2.42*
	ND	-.24	-1.79	.36	2.91**
	VPA/SSA * ND	.42	2.90**	.41	3.39***
Not worrying (NW)					
	VPA/SSA	.13	0.89	.19	1.38
	NW	-.05	-.34	-.07	-0.52
	VPA/SSA * NW	-.18	-1.24	-.22	-1.55
Attention regulation (AR)					
	VPA/SSA	.19	1.34	.15	1.01
	AR	-.27	-1.96	.21	1.47
	VPA/SSA * AR	-.10	-.73	-.18	-1.21
Emotional awareness (EA)					
	VPA/SSA	.07	.48	.03	0.19
	EA	-.31	-2.30*	.03	0.20
	VPA/SSA * EA	-.20	-1.40	-.39	-2.70*
Self-regulation (SR)					
	VPA/SSA	.02	0.11	.08	0.62
	SR	-.16	-1.20	.02	0.16
	VPA/SSA * SR	-.33	-2.21*	-.43	-3.31**
Body listening (BL)					
	VPA/SSA	.08	0.56	.03	0.21
	BL	-.18	-1.32	-.12	-0.83
	VPA/SSA * BL	-.26	-1.83	-.37	-2.52*
Trusting (TR)					
	VPA/SSA	.04	0.25	.03	0.18
	TR	-.05	-0.33	-.02	-0.14
	VPA/SSA * TR	-.30	-1.98	-.42	-3.01**

* *p* < .05

** *p* < .01

*** *p* < .001, not corrected for multiple testing

^a^ Significant interaction effects suggest that this domain of interoceptive awareness impacts or is related to sexual arousal concordance

#### Heartbeat perception

Heartbeat perception was not a significant predictor of genital arousal, subjective arousal, or sexual concordance.

#### Noticing

**Fig 1:**
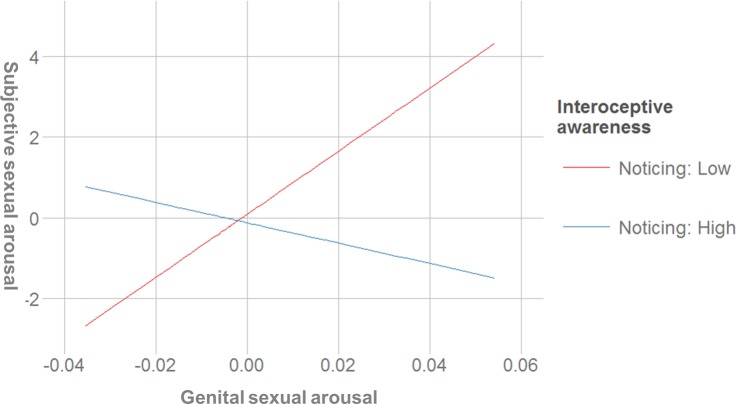
Interaction between subjective sexual arousal and the noticing scale for the prediction of genital sexual arousal (centered variables).

The interaction between noticing and subjective as well as genital arousal was a negative predictor of genital and subjective arousal, respectively. In other words, noticing was related to *lower* sexual arousal concordance. A post hoc simple slopes analysis indicated that the slope of the line that described the relationship between subjective and genital arousal for women that scored lower on the noticing scale (-1 *SD*) was positive (*b* = 42.48, *SE* = 11.61, *t* = 3.66, *p* < .001), while the slope of the line for women that scored high on that scale (+ 1 *SD*) was not different from zero (*b* = 5.85, *SE* = 14.43, *t* = -0.41, *p* = .687). In other words, subjective and genital sexual arousal were only positively associated in women with lower noticing scores (Fig 1).

#### Not-distracting

**Fig 2:**
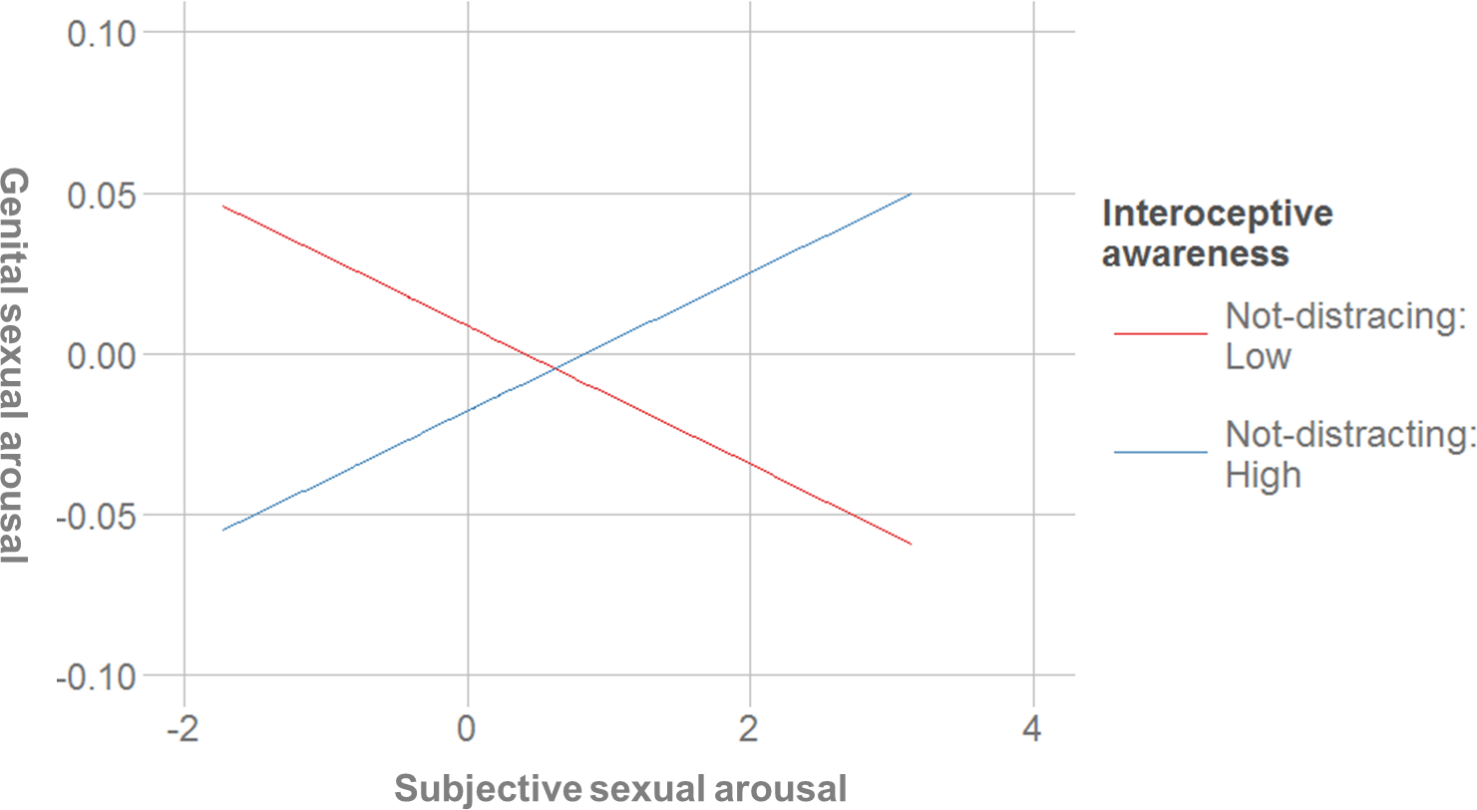
Interaction between subjective sexual arousal and the not-distracting scale for the prediction of genital sexual arousal (centered variables).

This subscale was a positive predictor of subjective arousal and it was also predictive of greater sexual concordance. A post hoc simple slopes analysis indicated that the slope of the line that described the relationship between subjective and genital arousal for women that scored lower on the not-distracting scale (-1 *SD*) was not significantly different from zero (*b* = -.012, *SE* = .01, *t* = -1.83, *p* = .074), while the slope of the line for women that scored high on that scale (+ 1 *SD*) was significantly positive (*b* = .010, *SE* = .004, *t* = 2.60, *p* = .012). In other words, subjective and genital sexual arousal were only positively associated in women with higher non-distracting scores (Fig 2).

#### Emotional awareness

**Fig 3:**
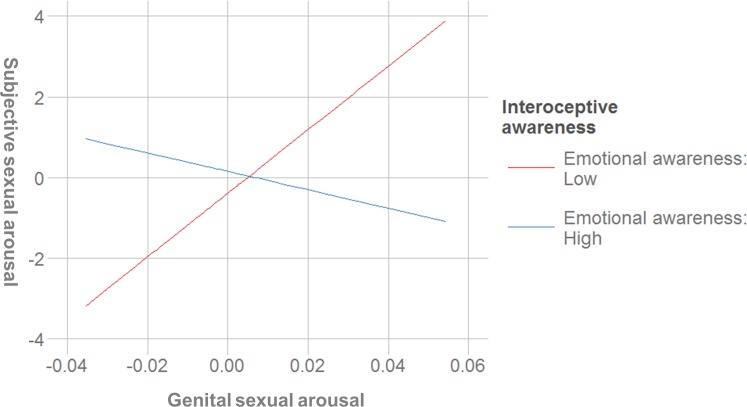
Interaction between genital sexual arousal and the emotional awareness scale for the prediction of subjective sexual arousal (centered variables).

This scale was a negative predictor of genital sexual arousal, indicating that women’s greater emotional awareness was associated with lower VPA. In addition, emotional awareness was a negative predictor of sexual arousal concordance. A post hoc simple slopes analysis indicated that the slope of the line that described the relationship between subjective and genital arousal for women that scored lower on the emotional awareness scale (-1 *SD*) was positive (*b* = 45.77, *SE* = 11.95, *t* = 3.83, *p* < .001), while the slope of the line for women that scored high on that scale (+ 1 *SD*) was not different from zero (*b* = -5.67, *SE* = 13.98, *t* = -0.41, *p* = .687). In other words, significant sexual concordance was only found in women with lower emotional awareness scores (Fig 3).

#### Self-regulation

This scale was a negative predictor of sexual arousal concordance. A post hoc simple slopes analysis indicated that the slope of the line that described the relationship between subjective and genital arousal for women that scored lower on the self-regulation scale (-1 *SD*) was positive (*b* = 50.80, *SE* = 12.80, *t* = 3.98, *p* < .001), while the slope of the line for women that scored high on that scale (+ 1 *SD*) was not different from zero (*b* = -16.80, *SE* = 17.40, *t* = -0.97, *p* = .338). In other words, significant sexual concordance was only found in women with lower self-regulation scores.

#### Body-listening

This scale was a negative predictor of sexual concordance. The pattern of results is similar to the emotional awareness and the self-regulation scale. The post hoc simple slopes analysis indicated that the slope of the line that described the relationship between subjective and genital arousal for women that scored lower on the body-listening scale (-1 *SD*) was positive (*b* = 50.14, *SE* = 14.02, *t* = 3.58, *p* < .001), while the slope of the line for women that scored high on that scale (+ 1 *SD*) was not different from zero (*b* = -8.65, *SE* = 15.59, *t* = -0.55, *p* = .582).

#### Trusting

The trusting scale was also a negative predictor of the association between subjective and genital sexual arousal. For this scale, the post hoc simple slopes analysis showed once again that the slope of the line that described the relationship between subjective and genital arousal for women that scored lower on the trusting scale (-1 *SD*) was positive (*b* = 48.72, *SE* = 12.92, *t* = 3.77, *p* < .001), while the slope of the line for women that scored high on that scale (+ 1 *SD*) was not different from zero (*b* = -7.76, *SE* = 14.32, *t* = -0.54, *p* = .590).

## Discussion

The goal of this study was to examine the association between interoceptive awareness and sexual concordance in a sample of women with low sexual desire.

The heartbeat perception task asked women to estimate the number of their heartbeats during three periods of time with a total duration of 105 seconds. The estimated number of heartbeats in relation to the actual number was used as a measure of interoceptive awareness. Overall, women were approximately 61% accurate in their estimation of heart rate. The accuracy with which women were able to estimate their heartbeats was not associated with sexual arousal concordance in our sample of women with SIAD. These findings are in line with the findings of others who similarly found no association between heartbeat accuracy and concordance in sexually healthy anxious and non-anxious women [32].

Given that we assessed heartbeat accuracy during an unaroused state, this may explain why we failed to find an association between this measure of interoceptive awareness and sexual arousal concordance, given that the latter was calculated during a period of heightened sexual arousal. Because these women were not aroused, this may have rendered it more difficult to perceive their heart rate, potentially resulting in women guessing their heart rates. Interestingly, heartrate accuracy varied greatly among our sample (from 0–0.96 out of a maximum possible 1.0) with some participants missing their actual number by only four heartbeats. It may be that some women show a high degree of agreement between heart rate accuracy and sexual arousal concordance whereas other women do not. Future research should aim to identify the predictors of women who have a high level of association between these different indices of interoception.

Although a well-validated task [47], it is still possible that participants may have been biased by their expectancies of heartrate, time estimation ability, or mean heart rate level [48]. It has also been suggested that the lack of association between interoceptive awareness and sexual arousal concordance may be due to methodological factors [32]. For instance, a signal detection paradigm in which participants were asked if a light flash coincided with a heartbeat or stomach contraction yielded a significant correlation (*r* = .51, *p* < .05) between heartbeat perception and stomach contractions [30]. Thus, it is possible that a stronger correlation may have been found if a signal detection paradigm had been used; however, it is important to note that other signal detection studies have also failed to find a significant relationship between interoceptive tasks, such as heartbeat perception and respiratory resistance [32,49].

In contrast to the objective measure of interoception, several subscales on our self-report measure of interoceptive awareness were significantly related to sexual concordance in our sample of women with sexual concerns. Five lower order factors of the MAIA were negative predictors of sexual concordance. Women who scored high on the noticing, emotional awareness, self-regulation, body listening, and trusting domains showed *lower* sexual concordance than women who scored low on these domains. Given that all of these subscales inquired about general body awareness, and not body awareness specifically to sexual sensations, it may be that women who scored higher on noticing and body listening display a choiceless awareness such that their awareness expands to all areas of the body equally. Equanimity is a Buddhist term referring to paying attention with equal interest, and can be seen during mindfulness practice when an individual is equally likely to notice pleasant, unpleasant, and neutral sensations. As sexual arousal was increasing with the erotic film, it is possible that women with higher noticing and body listening scores paid as much attention to other non-sexual sensations in their body, as they paid to sexual sensations. In other words, as genital arousal increased, their subjective arousal did not show the same corresponding increase.

Only one scale was associated with *greater* sexual concordance: Women who scored high on the not-distracting scale, indicating that they do not avoid or distract themselves from pain or discomfort, showed higher sexual concordance. In addition, women that scored high on the noticing scale also reported greater subjective sexual arousal. In other words, women who do not distract themselves from uncomfortable feelings felt more aroused sexually during the film and had a greater agreement of subjective and genital arousal. Given studies that show an increase in negative affect despite the presence of subjective sexual arousal, it is possible that women with higher non-distracting scores were unaffected by these potential increases in negative affect, and their subjective sexual arousal increased in parallel with their physiological arousal. On the other hand, women with lower not-distracting scores may have been distracted by increases in negative affect. Future studies should aim to explore the role of elicited emotions during similar paradigms of interoceptive awareness and sexual concordance.

Based on the Chivers et al. [25] meta-analysis, we expected that women with SIAD would have a lower concordance score than the average of *r* = .26 previously found in healthy women, particularly because the meta analysis revealed sexually dysfunctional women to have an average concordance score of *r* = .04 [25]. However, our results indicated a similar concordance score (*r* = .27) to the average concordance score of sexually healthy women in the meta-analysis. Because very few studies have deliberately compared concordance between women with and without sexual dysfunction, it is still not clear whether sexual dysfunction is associated with lower sexual arousal concordance or not [50]. However, it is important to acknowledge that concordance scores have varied across studies, ranging from *r* = -.80 to *r* = .95, thereby reflecting the tremendous heterogeneity of sexual concordance across women, with or without sexual concerns [39]. Taken together with the findings of the previous meta-analysis, our study suggests that women with sexual dysfunction and sexually healthy women show considerable variability in their sexual concordance, and the predictors of this variability should be a focus of future study.

### Clinical implications

The results of this study have key clinical implications and provide insights as to the relevance of interoceptive awareness for women’s sexual response and sexual function. Mindfulness-based interventions are effective in treating sexual dysfunctions [e.g. 10] and include exercises that encourage participants to focus on body sensations in the present moment. Improving non-judgmental awareness of different bodily sensations (i.e., breathing) is one goal of mindfulness-meditation [50]. Interestingly, our findings suggest that the non-attachment to sensations, and equanimity that is fostered with mindfulness practice, may not necessarily enhance sexual arousal, and may even lower sexual concordance. One implication of this finding is that mindfulness meditation may enhance women’s ability to notice all body sensations, not just sexual ones, and therefore lead to reduced sexual concordance.

Women with greater interoceptive awareness related to the noticing, emotional awareness, self-regulation, body listening, and trusting domains may be more prone to paying attention to all sensations in the body which may detract from noticing the specifically sexual sensations.

These findings do not directly challenge the assumption that by improving mindfulness women that oftentimes feel distracted during sexual activity (e.g., by non-sexual thoughts, sexual concerns, or body image issues) can learn to stay more present and engaged during sexual activity. In line with this assumption, the not-distracting domain of interoceptive awareness was indeed predictive of higher subjective arousal as well as higher sexual concordance. To increase sexual arousal during sexual activity may be particularly relevant for women with SIAD, as a significant number of these women report a lack of sexual excitement or reduced feelings of sexual arousal, even though they experience no difficulties in getting physically aroused [51].

### Limitations and future directions

This study is limited by the sample size of 52 women, and although comparable to other studies, it still yields a relatively low statistical power. As this sample was recruited as part of a treatment study for sexual concerns, we do not know if our participants different from other women with sexual difficulties who did not seek treatment in variables relevant to our findings. As well, the ecological validity of our findings cannot be established given that sexual arousal in a laboratory setting may not generalize to sexual arousal in the real-life setting. The extent to which interoceptive ability may be impacted by the laboratory setting is also unknown, and it may be the case that women are better able to perceive bodily sensations when they are in their own naturalistic environments. Future studies should attempt to measure interoceptive awareness and sexual arousal in the at-home environment, as well continue to look into individual differences, such as sexual histories, attitudes, and responsiveness. Moreover, given that this is the first study examining the relationship between concordance and interoception among women with sexual dysfunction, future studies should aim to replicate the current design.

### Conclusion

While objectively measured heartrate awareness was unrelated to genital or subjective sexual arousal and concordance in our sample of women with low sexual desire, self-reported interoceptive awareness was significantly related to both outcomes. Five aspects of interoceptive awareness (noticing, emotional awareness, self-regulation, body-listening, and trusting), were predictive of *lower*, and one aspect (not-distracting) was predictive of *higher* sexual concordance. These findings provide additional nuance to understanding the mechanisms by which psychosocial interventions, such as mindfulness meditation, may impact sexual functioning and response in women. Specifically, the findings suggest that although mindfulness may be associated with greater ability to notice sensations and listen to the body, these may not translate into increases in sexual arousal concordance, but instead, may actually decrease sexual arousal concordance. As pointed out by Suschinsky and Lalumiere [37], one must not be led to conclude that low sexual concordance, or even sexual discordance, is indicative of a dysfunction, and in fact, it may represent the norm rather than aberration. Clearly, more research is needed to understand what the meaning is behind high or low levels of sexual arousal concordance in women, and how this relates to their awareness of other non-sexual physiological signs.

## Supporting information

S1 Dataset (SAV)
