# Correction: Interoception and sexual response in women with low sexual desire

**DOI:** 10.1371/journal.pone.0203094

**Published:** 2018-08-23

**Authors:** Julia Velten, Lori A. Brotto

A reference is omitted from the second sentence of the third paragraph under the subheading “Interoceptive awareness and sexual concordance” in the Introduction section.

The sentence should read: In a sample of 26 women, the noticing subscale of the Multidimensional Assessment of Interoceptive Awareness scale (MAIA) [34] was related to greater sexual concordance (Handy et al., 2016).

The reference is: Handy AB, Meston CM. Interoceptive awareness moderates the relationship between perceived and physiological genital arousal in women. J Sex Med. 2016;13:1907–1914. https://doi.org/10.1016/j.jsxm.2016.09.018
